# The Neurocognitive Effects of Ghrelin-induced Signaling on the Hippocampus: A Promising Approach to Alzheimer’s Disease

**DOI:** 10.7759/cureus.3285

**Published:** 2018-09-11

**Authors:** Robert S Seminara, Charan Jeet, Sharmi Biswas, Bushra Kanwal, Waleed Iftikhar, Md Sakibuzzaman, Ian H Rutkofsky

**Affiliations:** 1 Neuroscience, California Institute of Behavioral Neurosciences & Psychology, Fairfield, USA; 2 Research, California Institute of Behavioral Neurosciences & Psychology, Fairfield, USA; 3 Pediatrics, California Institute of Behavioral Neurosciences & Psychology, Fairfield, USA; 4 Department of Research, California Institute of Behavioral Neurosciences & Psychology, Fairfield, USA; 5 Internal Medicine, CMH Lahore Medical College and Institute of Dentistry, Lahore, PAK; 6 Medicine, International American University College of Medicine, Washington, D.C., USA

**Keywords:** ghrelin, hippocampus, alzheimer's, neurocognitive, memory, growth hormone secretagogue receptor, ghs-r

## Abstract

The communication between the gastrointestinal tract and the central nervous system (CNS) allows for certain peptide hormones to influence neurocognitive function. Ghrelin, also known as the ‘hunger hormone,' has the unique ability to enter the CNS and interact with the growth hormone secretagogue receptor (GHS-R) within the hippocampus. Upon interaction with ghrelin, a conformational change in the receptor causes an increase in transcription factors to foster a wide array of physiologic changes in response to caloric deprivation. With the GHS-R in a relatively high concentration within the hippocampus, ghrelin can promote memory, spatial, learning, and behavioral effects. In fact, ghrelin appears to also have a neuroprotective and neuromodulatory response once active within the hippocampal dentate gyrus. Through the GHS-R, higher levels of ghrelin may alter cognitive circuitry and offer a possible link to the treatment of some pathologies implicated in neurological dysfunction. Alzheimer’s disease (AD) is already becoming a significant target for ghrelin neuroreceptor therapy. In such experimental models, ghrelin has been shown to combat this degenerative process by eliciting an ameliorative and regenerative response. Although trials and research are still ongoing, further studies are indicated as early research into this adjuvant therapy is promising.

The research team explored the effects of ghrelin by reviewing the downstream signaling modifications of ghrelin's interaction with a specific CNS receptor, the GHS-R. Although the GHS-R is found in multiple locations within the CNS, the team investigated the role of the GHS-R within the hippocampus to focus solely on the neurocognitive implications of ghrelin. The team noted which signaling pathways in particular that ghrelin initiated and used this approach to determine whether ghrelin may have any therapeutic benefits. The team explored the possible therapeutic indications of ghrelin by looking at studies conducted with a specific neurodegenerative disease known to target the hippocampus.

## Introduction and background

In order to fully understand the architecture of neurocognition, it is imperative to map the signaling pathways and effects of certain metabolic hormones active within the central nervous system (CNS). One hormone, in particular, is worth further exploration as its receptor utilizes multiple signaling pathways responsible for both metabolic and neurologic functionality [1]. Ghrelin is a unique peptide hormone produced in the gastrointestinal tract with a documented impact on the regulation of energy expenditure and neuronal protection [2]. Blood levels of ghrelin tend to increase in the fasting state, suggesting a primary regulatory response in different metabolic conditions [3]. Ghrelin is of particular interest as it may freely cross the blood-brain barrier and influence both synaptic and structural plasticity of certain parts of the brain [4-5]. The hippocampus, known for its role in cognitive function through the formation of active and recall memories, is a crucial part of neurocognitive studies since it is home to a variety of neurologic receptor signaling pathways [6]. Learned memories, behaviors, and spatial reformative sequences all have a link to the hippocampus [7-8]. The hippocampus also has a high susceptibility to damage in multiple neurodegenerative diseases. It is the role of neurocognitive studies to investigate cognitive ability by mapping specific tasks or behaviors to distinct areas or circuits within the brain. The purpose is to provide a more precise picture of the relationship between physiology and psychology, connecting each system at a molecular level. In order to focus on the impact of ghrelin, its endogenous ligand, the growth hormone secretagogue receptor (GHS-R), will be of great importance [9]. The GHS-R plays a unique role in the hippocampus, and it may indeed be the best tool to measure the signaling cascade initiated by ghrelin.

Our goal is to explore the neurocognitive impact of ghrelin and report its mechanism of action through various signaling pathways within the hippocampus. The fluctuating levels of ghrelin in the bloodstream may be clues to the subtle fluctuations in cognitive ability throughout the day and over the course of years. Ghrelin and other neuropeptides may open the door to treatment of some debilitating neurocognitive diseases. Metabolic manipulation treatment through neuropeptides is under careful consideration as neurodegeneration appears to grow alongside changes in metabolism and advanced age [10]. Ultimately, the full implications of ghrelin’s reach into the CNS will continue to be unveiled through subsequent studies and trials. For this article, we focused our search on the GHS-R typically found in high concentration in the hippocampus [11].

## Review

The biochemical map for neurogenesis and cognitive function appears to be interlinked with hormones that fluctuate over time. Ghrelin is one part of the gastrointestinal feedback system known to regulate hunger in accordance with daily productivity [12]. With increasing studies uncovering a link between ghrelin and cognitive function, there is a need for further exploration of the possible therapeutic benefits. As such, it is essential to review the neuronal signaling of ghrelin and its downstream effects. As research continues to build a framework for understanding ghrelin’s behavior and corresponding receptors, there is significant urgency for increased testing with adjuvant therapy regimens. Ultimately, the results may be a cost-effective, unyielding breakthrough in the neurocognitive treatment model for diseases such as Alzheimer's.

Physiologic properties of ghrelin

The connection between the CNS and the enteric nervous system (ENS) relies on the dissemination of peptide hormones produced in the gastrointestinal tract. One such neuropeptide with the unique ability to penetrate the blood-brain barrier while originating within the lining of the stomach and pancreas is ghrelin [13]. Encoded via the GHRL gene, ghrelin is a 28-amino acid Ser3 acylated peptide believed to be responsible for inducing hunger and maintaining different levels of homeostasis [13]. Ghrelin uses a wide array of signaling pathways to achieve this level of dialogue with the CNS. For instance, evidence suggests ghrelin has an impact on the mammalian target of rapamycin (mTOR) signaling pathway; mTOR is remarkable for its role in cancer, diabetes, depression, and obesity. Early studies have shown a response in this pathway to circulating levels of ghrelin [14]. The orexigenic mechanism of ghrelin also works through alterations of sirtuin 1 (SIRT1), p53, and adenosine monophosphate-activated protein kinase (AMPK); the triggered response is an increase in the agouti-related protein (AgRP) and neuropeptide Y (NPY) in the arcuate nucleus of the hypothalamus [15].

Various animal studies using mice continue to reveal the downstream transcription and modification by ghrelin while dose-response analytical trials elude to this hormone's extensive role in the CNS [1, 16-17]. Ghrelin uses a seven transmembrane (7TM) receptor known to contribute to various physiological functions which regulate growth hormone secretion, lipolysis, and caloric intake, as well as dopaminergic and cognitive ability [1]. Logically, this would make the growth hormone secretagogue receptor (GHS-R) the next step in understanding ghrelin’s neurophysiological impact as it initiates pathways in the hippocampus.

Activation of the GHS-R

The GHS-R is a G-protein-coupled receptor with a markedly high catalytic activity in the brain [2]. Not until recently was the GHS-R complex thought to control anything further than metabolic regulation. The growth hormone secretagogue receptor type 1a (GHSR1a), in particular, has been found in multiple regions of the brain which suggests its role beyond neurocognitive functionality [18]. However, the messenger ribonucleic acid (mRNA) of GHSR1a was found in elevated concentrations within the dentate granular layer in mice, suggesting a role in hippocampal neuronal homeostasis. When ghrelin-O-acyltransferase (GOAT) acetylates ghrelin, it was then capable of interaction with the GHSR1a. This functional relationship now enables ghrelin to advance its reach into the CNS.

The hippocampus is a vital part of the limbic system known for its integral role in various aspects of learning, spatial knowledge, and memory [19]. Metabolic regulatory factors, such as insulin-like growth factor 1 (IGF-1), glucagon-like peptide 1 (GLP-1), and ghrelin, all seem to work in tandem at creating a dialogue between the enteric and central nervous system [20]. The structural integrity of hippocampal neurons may even be incumbent on such activity. In mapping out the neuronal layout of the hippocampus, multiple studies have found the GHS-R within the dentate gyrus, as well as the Cornu Ammonis (CA1 and CA2) [21]. Evidence suggests the release of ghrelin triggers a protective mechanism on dendritic spinal density, thereby preserving the structural integrity of the hippocampus [21-22]. According to a recent study, impaired ghrelin signaling (tested through GHS-R knockout mice) has even led to advanced aging of the hippocampal dentate gyrus [21].

The ghrelin receptor GHSR1a bares a strong a connection with cognitive function, particularly when the gene encoding this receptor is altered. In conjunction with dopamine receptors (DRD1a and DRD2), the association between ghrelin with learned behavior/memory seems even more apparent [23]. However, the effect on cognitive ability may be more of a modulatory function as opposed to a direct alteration. Once ghrelin binds to GHSR1a, a signaling cascade ensues as dopamine and glutamate are released, followed by an accelerated rise in cyclic adenosine monophosphate (cAMP) [24-25]. The causal link has yet to be fully established or understood regarding neuromodulation, although this reoccurring sequence appears to be consistent in animal studies. Interestingly, one study with knockout mice for GHSR1a (labeled GHSR -/-) showed increased spatial memory, yet impaired contextual memory [23]. The researchers believed this to point again toward the neuromodulatory type function of ghrelin which may depend on other neurotransmitters, ultimately to create a cascade of long-term potentiation.

In 2015, Ghersi et al. found the activation of the GHS-R1a and the release of glutamate to be the attributable mechanism for understanding how ghrelin plays a role in cognitive function [26]. In the presynaptic terminal within the hippocampal neurons, an influx of calcium enables the release of glutamate and subsequently facilitates the expression of the N-methyl D-aspartate receptor subtype 2B (NR2B). The NR2B is of importance here, as it has been shown to empower memory consolidation and combat the effects of Ro 25-6981 (a known impairment marker in mice for long-term memory consolidation) [26]. Once again, there is a strong neurocognitive association of ghrelin beginning at the G protein-coupled receptor (GPCR) level. Nevertheless, the challenge remains in verifying these changes in humans at both the molecular and psychological level. The widespread signaling processes reveal why more studies are warranted to track the full effects of either ghrelin supplementation or deprivation.

Cognitive performance quantified

There is an ongoing debate regarding the reciprocal relationship between ghrelin and cognitive performance scores in humans. While animal studies tend to show a positive effect on hippocampal neurons, human studies reveal something different [27]. For instance, in a lab model using mice, a study was designed to test the role of the ghrelin’s involvement in hippocampal spatial cognitive function. Administration of systemic ghrelin for eight days did not alter spatial cognitive performance, yet it stimulated adult neurogenesis [28]. Similarly, a previous study in 2012 found the GHS-R1a deficient mice showed better spatial memory tests with worsening contextual fear type conditioning [23]. To make ghrelin’s impact even more elusive, a 2016 human study found no changes in a cognitive battery test following the acute administration of ghrelin [24]. Although ghrelin may modulate the functional activity of the brain, the subjects in this test failed to show any changes in short-term memory [24]. In 2010, ghrelin levels were tested in a group of non-demented elderly participants. The study found that there was an inverse relationship to cognitive performance and levels of ghrelin [27]. Although this study was limited to 35 participants, what accounts for the disparity among each individual test? Is ghrelin more of a long-term neuromodulatory hormone? Should studies be examining the concurrent signaling effects and cognitive battery tests of local or systemic ghrelin administration?

Perhaps using long-term dietary restriction may be another way to assess if endogenous ghrelin may be more effective. In a study which predisposed mice to three months of caloric deprivation, there was a marked increase in the survival of hippocampal neurons [16]. This result was not seen in GHS-R1a knockout mice, revealing a pivotal role of GHS-R1a and its influence on dendritic neuronal health [29]. The shared component of the positive effects in animal models appears to be long-term treatment with ghrelin or chronic exposure to caloric deprivation [17]. This is not to say the only signaling pathway in controlling neurogenic stem cell proliferation is via GHS-R1a; mitogen-activated extracellular signal-regulated kinase (MEK/ERK1/2), phosphatidylinositol-4,5-bisphosphate 3 kinase/protein kinase B/glycogen synthase kinase 3 beta (PI3K/Akt/GSK-3β), and Janus kinase 2/signal transducer and activator of transcription protein 3 (JAK2/STAT3) have all been implicated in mice for having neuronal protection and proliferation mediated through ghrelin release [16]. Ghrelin even reaches so far as having a cyclin A and cyclin-dependent kinase (CDK) 2 in the hippocampus [30]. With the ability to impact the cell cycle through the above mechanisms, using ghrelin's physiologic properties as a possible treatment for unregulated cell processes and pathologies is warranted.

Targeting a neurological disease

The key to understanding how neurocognitive architecture utilizes multiple hormones and signaling pathways may involve targeting a disease. Alzheimer’s disease (AD) is known for its attack on the cerebral cortex and other subcortical regions of the brain, typically affecting people above the age of 65 [31-32]. The number of people afflicted with the disease is expected to nearly triple by 2050, while one in 10 people in the United States age 65 and older already have some form of Alzheimer's dementia [33-35]. Alzheimer’s is also the sixth leading cause of death in the United States and healthcare costs are expected to reach 1.1 trillion dollars by 2050 [33-35]. Although many hypotheses exist to try and locate the precise pathophysiology of AD (i.e., beta-amyloid toxicity, tau proteins, mitochondrial dysfunction, synaptic destruction), the reoccurring postulations appear to be the involvement of several molecular signaling pathways [36]. Similar to the cascade that follows activation by ghrelin, AD may share a common etiology. Within the past five years, ghrelin has already begun to show some promise with therapeutic application in multiple AD research trials (see Table 1).

**Table 1:** Relevant Studies From 2013-2018 Showing the Therapeutic Benefits of Ghrelin with Alzheimer’s Disease Morphology Aβ: amyloid beta; AβPP: amyloid-β protein precursor; AChE: acetylcholinesterase; AD: Alzheimer's disease; CNS: central nervous system; GHRP-6: growth hormone-releasing peptide 6; GHSR-1a: growth hormone secretagogue receptor type 1a; MK-0677: ibutamoren mesylate; pCREB: phosphorylated adenosine 3'5' cyclic monophosphate-response element binding protein

Author(s), Year	Country	Treatment	Findings	Summary
Eslami et al., 2018 [37]	Iran	Intracerebrovaslcular ghrelin administered for two weeks	Male Wistar rats with induced Aβ1-42-induced neurotoxicity showed improvement of memory retention and alleviation of memory loss through relief of the Aβ1-42-induced synaptic plasticity destruction	Chronic ghrelin administration showed therapeutic benefit with Alzheimer's disease-like pathology.
Jeong et al., 2018 [38]	Korea	MK-0677, a ghrelin agonist which activates GHSR-1a	MK-0677 treated mice showed reduced Aβ deposition, gliosis, and neuronal and synaptic loss in the deep cortical layers; the treatment also blocked the reduction of pCREB levels in the dentate gyrus of the hippocampus.	The ghrelin agonist MK-0677 may be an effective treatment for early-stage AD.
Santos et al., 2017 [39]	Portugal, United Kingdom, Brazil, Australia	Acyl ghrelin injections for a seven-day duration	Ghrelin knockout mice and Aβ1-40 treated mice showed improved recognition and spatial memory after treatment with acyl ghrelin for seven days.	Acyl ghrelin may slow progression of AD and may delay the onset of early AD.
Cecarini et al., 2016 [40]	Italy, United States	Ghrelin	Ghrelin showed a growth-promoting effect on neuronal cells via two intracellular proteolytic pathways: the ubiquitin-proteasome system (UPS) and autophagy; cells were of 2 types; AβPP gene or the 717 valine-to-glycine AβPP-mutated.	Neuronal homeostasis may be controlled by ghrelin through proteolytic pathways potentially involved in AD.
Kang et al., 2015 [41]	Korea	Intracerebroventricular acylated-ghrelin (AD-G) (0.2 nmol/h) and DES-acetylated ghrelin (AD-DES-G) (0.2 nmol/h)	AD-G worked to reduce amyloid deposition and decreased the phosphorylation of Tau protein; AD-G prevented a loss in cognitive function compared to the control.	Intermittent fasting may increase endogenous acyl-ghrelin levels which may have cognitive and metabolic' benefits, particularly in the early stages.
Kunath et al., 2015 [3]	Germany, United States	High glycemic index (GI) diet + ghrelin agonist	The high GI + ghrelin agonist treated mice had the best score on the water maze which represented memory performance. However, the ghrelin agonist did not affect the Aβ plaque load in the dentate gyrus of the hippocampus.	Ghrelin may provide therapeutic cognitive benefits in AD due to a CNS insulin signaling mechanism.
Madhavadas et al., 2014 [42]	India	Ghrelin receptor analog [D-Lys (3)] GHRP-6	Treatment with the ghrelin receptor analog reduced hippocampal Aβ and AChE levels, along with improved scores on the ‘Barnes maze’ task, as compared to controls.	The use of a ghrelin analog to reverse some pathologic mechanisms of AD may be of significant therapeutic benefit.
Moon et al., 2014 [43]	Korea	Peripherally administered ghrelin	AD model mice (5XFAD) showed increased neurogenesis with peripherally administered ghrelin; control mice showed impaired neurogenesis, although the Aβ region appeared to be the same in both groups.	Ghrelin may increase neurogenesis in AD without directly altering the Aβ pathology.
Gomes et al., 2014 [44]	Portugal	Ghrelin and leptin	The mHypoE-N42 cell line showed reduced Aβ toxicity (such as superoxide production, calcium rise, and mitochondrial dysfunction) with ghrelin supplementation.	Metabolic hormones, such as leptin and ghrelin, may have preventative implications for hypothalamic AD modifications.
Dhurandhar et al., 2013 [45]	United States	Long-term administration of ghrelin agonist LY444711	Long-term treatment with a ghrelin agonist has a similar neurocognitive benefit to caloric restriction in AD model mice.	Neuroendocrine, metabolic hormones affect age-related cognitive decline.

The studies discussed indicate a multifaceted approach to using ghrelin as a means to treat Alzheimer's disease. While some studies do not report alterations in the beta-amyloid levels in the hypothalamic nuclei and hippocampal dentate gyri, beneficial neurocognitive effects appeared consistent throughout.

Limitations

The limitations of assessing the neurocognitive benefit of one hormone within the limbic system can be extensive. One challenge is designing a proper research trial to fully and accurately assess a measurable outcome. Another challenge falls on isolating any confounders, as high or low levels of ghrelin often imply comorbidity with gastrointestinal pathology. Since ghrelin is produced in large quantities in the stomach, Helicobacter pylori positive patients have shown decreased plasma levels of circulating ghrelin [4]. Therefore, in conducting a clinical trial, selecting the appropriate subjects requires extensive testing. Innovative measures regarding ghrelin therapy should also be taken into consideration. By using different treatment modalities, researchers may find more comparable results compared to what has already been measured in mice. For instance, testing with a ghrelin agonist or reuptake inhibitor may provide sufficient levels capable of producing a short-term cognitive benefit. In contrast, using caloric deprivation to increase endogenous levels of ghrelin may not be adequate to produce a similar quantifiable outcome (in the short term). Ultimately, additional human studies with a wide array of treatment regimens may offer the greatest insight into the full neurocognitive potential of ghrelin.

## Conclusions

Based on the available data by search parameters, the research team concluded that ghrelin has a neurocognitive effect by way of the GHS-R within the hippocampus. Furthermore, ghrelin has shown the ability to interact with multiple signaling pathways within the hippocampal dentate gyrus. Additional investigation with a meta-analysis of patients placed on long-term supplemental ghrelin therapy and caloric restrictive diets may provide the best insight into the neurocognitive effects of ghrelin. Nevertheless, these preliminary results in mice may be of great importance to patients battling a neurological disease, such as Alzheimer's disease. This review, in particular, warrants further investigation into the potential therapeutic benefits and implications of ghrelin as a neurocognitive agent.
